# Thymus and lung mucosa-associated lymphoid tissue lymphoma with adenocarcinoma of the lung: a case report and literature review

**DOI:** 10.1186/s12957-023-02904-2

**Published:** 2023-01-23

**Authors:** Yu Pang, Daosheng Li, Yiqian Chen, Qinqin Liu, Yuheng Wu, Qingliang Teng, Yuyu Liu

**Affiliations:** 1 Department of Pathology, the Affiliated Taian City Central Hospital of Qingdao University, Tai’an, 271000 China; 2 Department of Rehabilitation, the Affiliated Taian City Central Hospital of Qingdao University, Tai’an, 271000 China; 3 Department of Hematology, the Affiliated Taian City Central Hospital of Qingdao University, Tai’an, 271000 China; 4 Department of Medical Imaging, the Affiliated Taian City Central Hospital of Qingdao University, Tai’an, 271000 China

**Keywords:** MALT lymphoma, Lung adenocarcinoma, Collision tumor, Sjögren’s syndrome, Case report

## Abstract

**Background:**

Mucosa-associated lymphoid tissue (MALT) lymphoma is a common, low-grade, malignant B-cell lymphoma. However, simultaneous MALT lymphoma in the thymus and lung is extremely rare, and concomitant adenocarcinoma of the lung is even rarer. Herein, we report a rare case of a collision tumor in which MALT lymphoma was found in both the thymus and lung with Sjögren’s syndrome (SS) and adenocarcinoma in the lung.

**Case presentation:**

A physical examination of a 32-year-old woman revealed an anterior superior mediastinal space-occupying lesion, and chest computed tomography (CT) indicated a nodular ground-glass opacity and irregular mixed-density focus in the right lung. All lung cancer-related tumor biomarkers were within normal ranges. The thymus and part of the lung tissue were surgically resected. The histopathology and molecular examinations confirmed MALT lymphoma of the thymus and lung with lung adenocarcinoma. SS was also diagnosed. No special postoperative treatment was performed for the MALT lymphoma, and the patient underwent immunosuppressive therapy for SS after 4 months of follow-up observation.

**Conclusions:**

MALT lymphoma of the thymus and lung tissues has no specific presentation on imaging and is difficult to differentiate from common malignant tumors, and the definite diagnoses of these tumors are highly dependent on histopathological examination in combination with molecular testing and cytogenetics. SS may be an important potential condition for the occurrence of MALT lymphoma in the thymus and lung. Additional similar cases are needed to clarify the biological pathways and potential molecular mechanisms of rare lymphomas and collision tumors.

## Background

Mucosa-associated lymphoid tissue (MALT) lymphoma is a low-grade, extranodal, B-cell lymphoma that usually develops in the gastrointestinal tract, parotid gland, and lung and accounts for approximately 7–8% of non-Hodgkin’s lymphomas [1, 2]. However, MALT lymphoma rarely involves the thymus, and it is even rarer for MALT lymphoma to emerge simultaneously in both the thymus and lung. While patients with lymphoma who have been treated with chemotherapy and/or radiotherapy have an increased probability of developing a second tumor, such dual primary malignancies arising in the same or adjacent organs are extremely rare and are termed collision tumors [3]. We describe for the first time a rare case of a collision tumor in the thymus and lung that presented with MALT lymphoma with Sjögren’s syndrome (SS) and concomitant adenocarcinoma of the lung.

## Case presentation

**Fig. 1:**
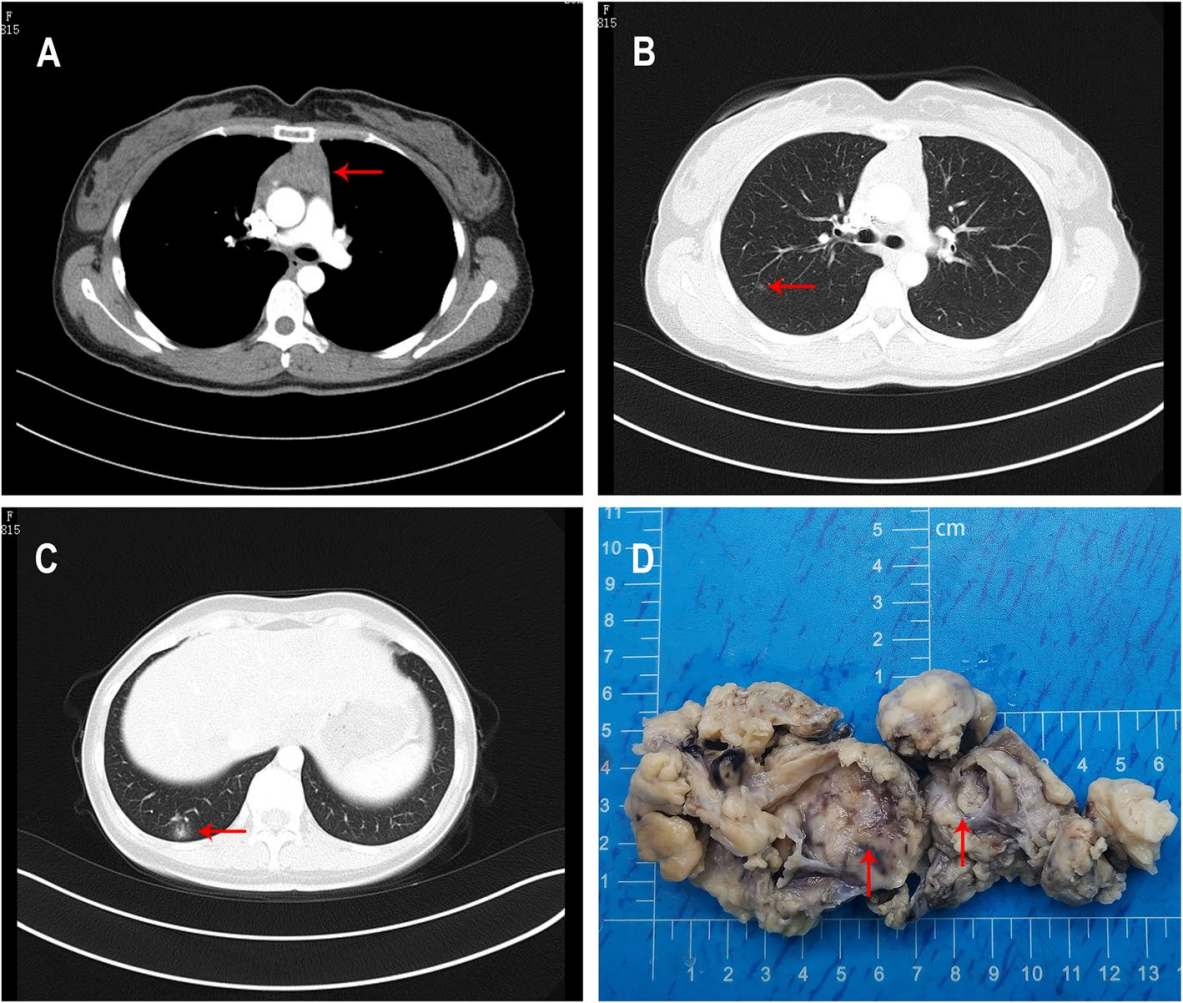
CT findings of the patient and macroscopic observation of the thymus specimen. Chest CT scan indicated a soft-tissue density opacity in the anterior superior mediastinal thymus region (**A** red arrow). Chest CT scan indicated a nodular ground-glass opacity in the posterior segment of the upper lobe of the right lung (**B** red arrow). Chest CT scan indicated an irregular mixed-density focus in the posterior basal segment of the lower lobe of the right lung (**C** red arrow). The thymus specimen was a cystic solid mass with a grayish-red cut surface and multiple small cystic cavities containing viscous fluid (**D** red arrow)

An asymptomatic 32-year-old woman with no previous medical history who denied a history of smoking was found to have an anterior mediastinal mass on physical examination. The complete blood count, liver and kidney function tests, and lung cancer-related tumor markers were all within normal ranges. Enhanced chest computed tomography (CT) indicated a soft-tissue density in the anterior superior mediastinal thymus region, measuring 4 cm × 3.5 cm in size with a cast shape, slight distension, clear margins, and uneven density (Fig. 1A). In addition, the enhanced CT indicated a nodular ground-glass opacity in the posterior segment of the upper lobe of the right lung, measuring 9 mm × 5 mm in size with clear margins (Fig. 1B) and an irregular mixed-density focus in the posterior basal segment of the lower lobe of the right lung, measuring 9.5 mm × 12.7 mm in size, adjacent to the pleura, with no significant retraction (Fig. 1C). No enlarged lymph nodes were found in the hilum or mediastinum. The results of the enhanced CT suggested the possibility of thymoma.

**Fig. 2:**
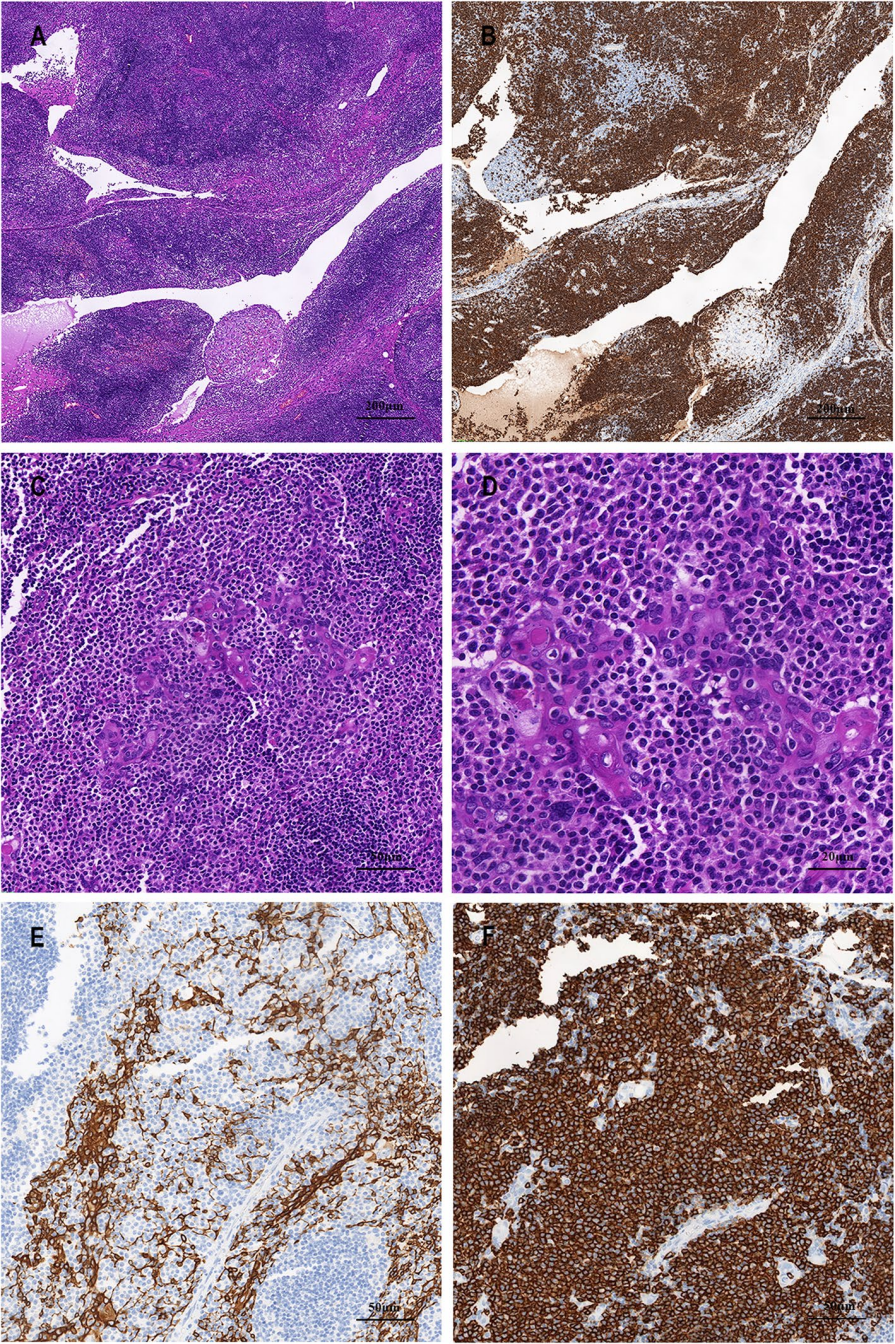
Histological examination and immunohistochemical staining of thymus. The thymus tissue was polycystic, and hyperplastic small lymphocytes had diffusely infiltrated the thymus tissue (**A** HE × 50). Immunohistochemistry (IHC) showed that hyperplastic small lymphocytes were positive for CD20 (**B** IHC × 50). Lymphoepithelial lesions (**C** HE × 200; D, HE × 400). IHC showed positive for CK5/6 in Hassall’s corpuscles and the thymic epithelium (**E** IHC × 200). IHC showed that mononuclear-like B cells invading the epithelium were positive for CD20 (**F** IHC × 200)

**Fig. 3:**
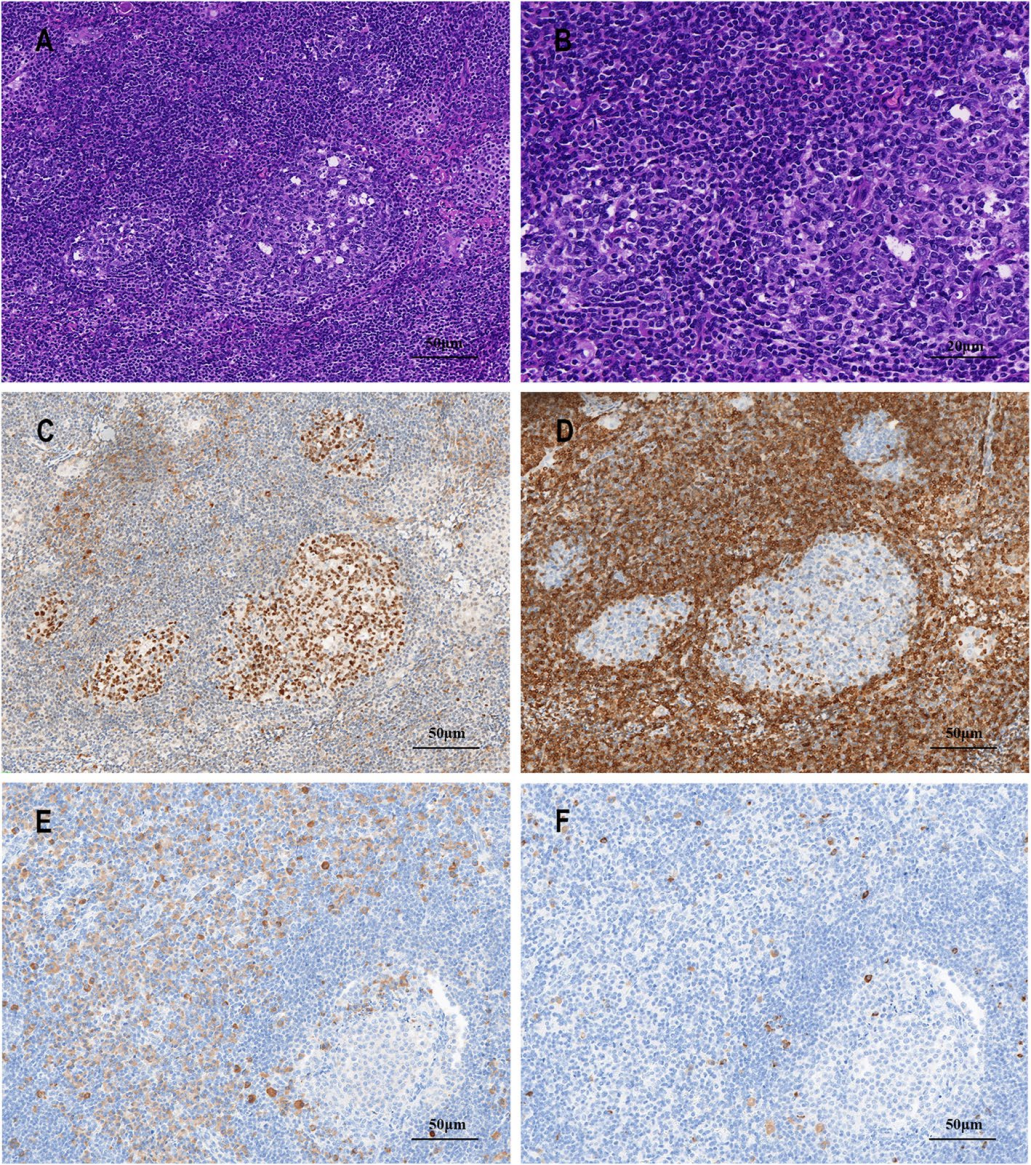
Lymphoid follicular colonization and IHC in thymus tissue. Follicular colonization (**A** HE × 200; **B** HE × 400). Germinal center was positive for BCL6 (**C** IHC × 200) and was negative for BCL2 (**D** IHC × 200). The staining results were positive for kappa light chain (**E** IHC × 200) and were negative for lambda light chain (**F** IHC × 200)

**Fig. 4:**
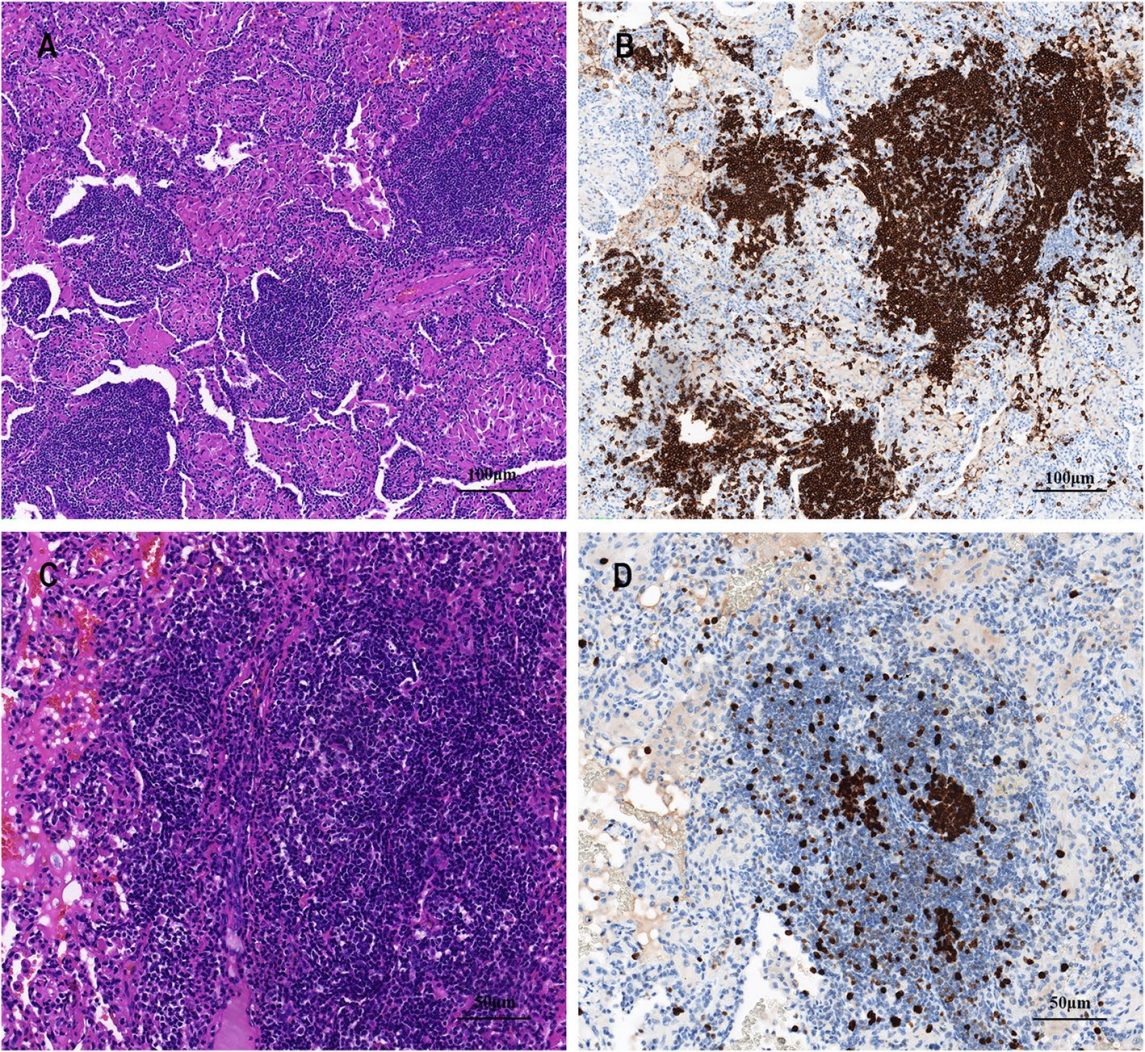
Lymphocytes infiltrated lung tissue and locally formed lymphoid follicles. A large number of small lymphocytes infiltrated in lung lesion (**A** HE × 100). IHC showed that the small lymphocytes were positive for CD20 (**B** IHC × 100). Lymphatic follicles formed in the local area of lung tissue (**C** HE × 200). Ki67 is highly expressed in germinal center (**D** IHC × 200)

**Fig. 5:**
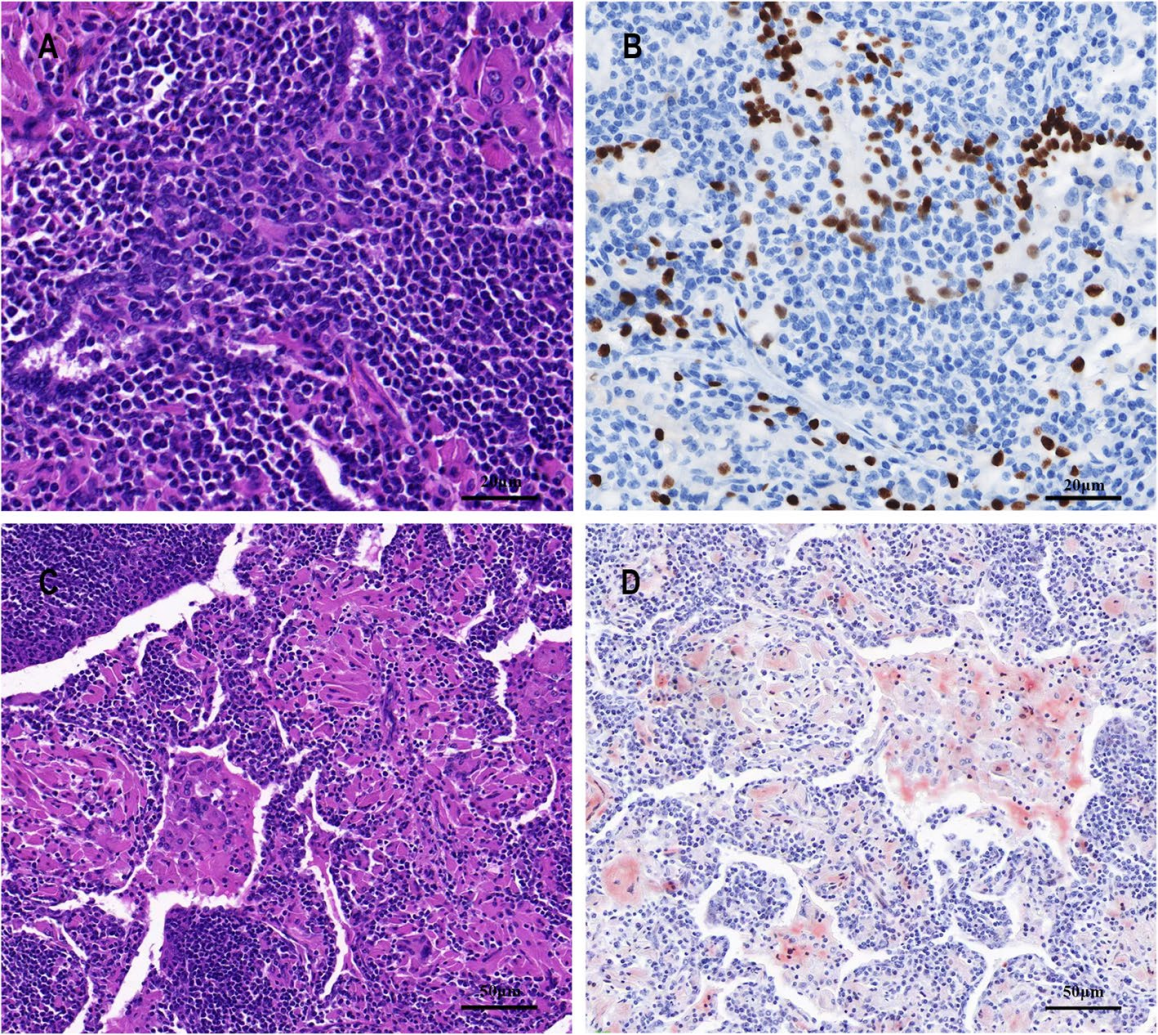
Small lymphocytes infiltrate bronchial gland epithelium and amyloidosis. The small lymphocytes invaded the bronchial gland epithelium to form lymphoepithelial lesions (**A** HE × 400). IHC showed that the bronchial gland epithelium was positive for TTF1, and the bronchial gland epithelium was destroyed by small lymphocytes (**B** IHC × 400). A large number of amyloid deposits were observed in the lung interstitium (C, HE × 200) and were positive for Congo red (**D** specific staining × 200)

**Fig. 6:**
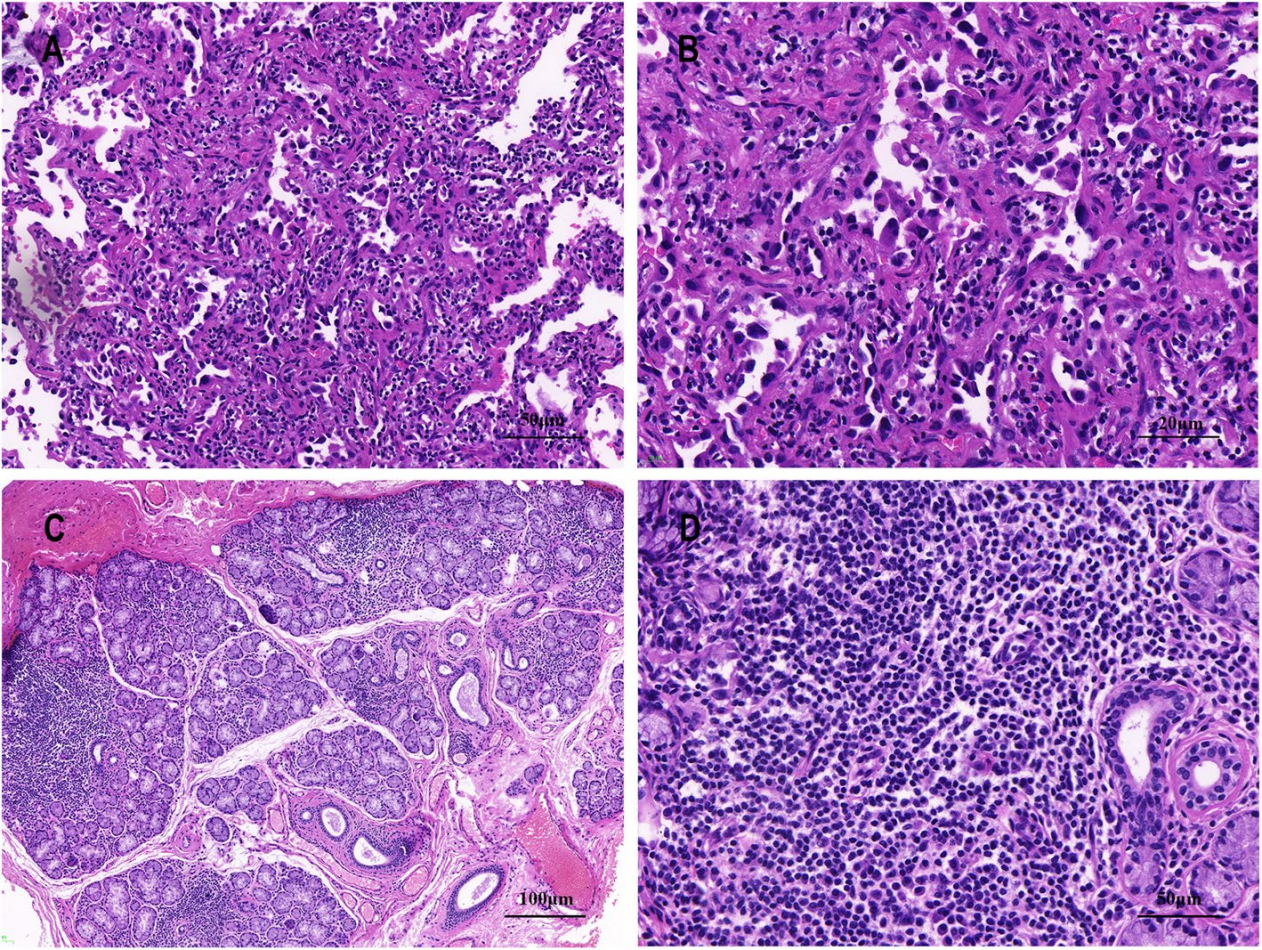
Histological examination of lung and labial glands. Microinvasive pulmonary adenocarcinoma (**A** HE × 200; **B** HE × 400). Labial gland biopsy (**C** HE × 100; **D** HE × 200)

The patient underwent a thoracoscopy-guided total thymectomy and wedge resection of the lung, and the resected specimen was observed visually. The thymus specimen was a cystic solid mass measuring 9 cm × 5 cm × 1.5 cm, with a grayish-red cut surface and multiple small cystic cavities containing viscous fluid (Fig. 1D). A 0.9-cm tough, gray-red nodule was found in the upper lobe of the right lung, and a 1.3 cm × 1.3 cm × 1 cm mass was found in the lower lobe of the right lung. Histopathological examination of the thymus under low magnification indicated destruction of the thymic structure, no obvious lobulated structures, and cystic formation. Pathological analysis revealed that the thymus tissue was polycystic under low magnification, and hyperplastic small lymphocytes had diffusely infiltrated the thymus tissue (Fig. 2A). Immunohistochemistry showed that hyperplastic small lymphocytes were positive for CD20 (Fig. 2B) and CD79a, positive for CD3 and CD5 in reactive proliferating T cells, and negative for TdT. Mononuclear-like B cells invaded Hassall’s corpuscles and the thymic epithelium and formed lymphoepithelial lesions (Fig. 2 C–D). Positive staining for CK5/6 was seen in Hassall’s corpuscles and the thymic epithelium (Fig. 2E). Immunohistochemistry showed that mononuclear-like B cells invading the epithelium were positive for CD20 (Fig. 2F). Tumor cells with infiltrating growth were seen around the follicles and invaded the mantle zone and follicles, leading to follicular colonization (Fig. 3 A–B). Immunohistochemistry showed that the germinal center was positive for BCL6 (Fig. 3C) and negative for BCL2(Fig. 3D). Positive staining for *κ* light chain (Fig. 3E) was observed in immunohistochemistry, but the specimens were negative for *λ* light chain (Fig. 3F). A final diagnosis of thymic MALT lymphoma was made. In the right lower lung lesion, a large number of small lymphocytes infiltrate were found in the lung tissue (Fig. 4A), and the small lymphocytes were positive for CD20 (Fig. 4B) and CD79a and negative for CD3 and CD5 in immunohistochemistry. Lymphatic follicles formed in the local area of lung tissue (Fig. 4C), and Ki67 is highly expressed in germinal center (Fig. 4D). Plasma cell differentiation was found in the interfollicular areas, and positive staining for CD38 was seen in the area of plasma cell differentiation. The small lymphocytes invaded the bronchial gland epithelium to form lymphoepithelial lesions (Fig. 5A) .The bronchial gland epithelium expressed TTF1, and the bronchial gland epithelium was destroyed by small lymphocytes (Fig. 5B). A large number of amyloid deposits were also observed in the lung interstitium (Fig. 5C), which stained positive for Congo red (Fig. 5D). Due to the atypical histopathological representation, additional polymerase chain reaction (PCR) gene scan of the lesions in the thymus and the right lower lung was performed, and both showed positive expression of immune globulin (Ig) HV and IgHD and negative expression of Igκ and Igλ. To understand the cytogenetic characteristics, we used fluorescence in situ hybridization (FISH) to detect anaplastic lymphoma kinase (ALK) break apart, MALT lymphoma-associated translocation 1 (MALT1) break apart, immunoglobulin heavy locus (IGH) break apart, and trisomy 3 of the diseased tissues in the thymus and lung. However, all results were negative. A final diagnosis of pulmonary MALT lymphoma was made. Moreover, the infiltrative growth of atypical glands was found in the lesions in the upper lobe of the right lung, and the diagnosis was microinvasive pulmonary adenocarcinoma according to the size of the lesion (Fig. 6 A–B). These findings confirmed the diagnosis of thymic and right lower lobe MALT lymphoma combined with microinvasive adenocarcinoma in the upper lobe of the right lung with no tumor at the surgical margin.

After further inquiring about the patient’s medical history, the patient revealed that she had experienced dry mouth symptoms for 5 years. The results for antinuclear antibody (1:1000) and SS-A/Ro antibody were positive. In the labial gland biopsy, lymphocyte and plasma cell infiltration were found in the interstitium with a multifocal distribution, with a plasma cell count > 50/foci (Fig. 6 C–D). The patient fulfilled the 2016 American College of Rheumatology/European League Against Rheumatism Classification Criteria for Primary SS [4] and was diagnosed with SS.

The patient underwent postoperative positron emission tomography/CT. According to the Lugano staging system [5], the patient was in stage 2E with no B symptoms, and she did not choose any special postoperative treatment for lymphoma. Follow-up observation was selected. The patient underwent immunosuppressive therapy for SS after 4 months of follow-up observation.

## Discussion and conclusions

Thymic MALT lymphoma originates from the mucosal tissue of the thymus. To date, 123 cases have been reported, with a high incidence in Asian populations and mostly in women [6, 7]. The majority of previously reported thymic MALT lymphomas have been cysts or cystic masses. Cysts are visible during gross thymectomy; however, cystic changes are not unique to thymic MALT lymphoma and can also be observed in thymoma, thymic carcinoma, mediastinal seminoma, and nonneoplastic thymic lesions [8]. When tumors grow within the thymus, cyst formation may be associated with a tendency toward a cystic transformation of the medullary duct epithelium [7]. The mixed presentation of epithelial components and small lymphocytes in thymic MALT lymphoma may be misdiagnosed as lymphocyte-rich thymoma. The epithelium can contain residual normal or hyperplastic thymic epithelial cells, but the lymphocytes in thymoma usually appear as small immature lymphocytes, with most exhibiting a T-cell immunophenotype. In contrast, the lymphocytes in thymic MALT lymphoma are B lymphocytes. In the present case, lymphoid follicle formation with dilated marginal areas and lymphoepithelial lesions was observed, and immunohistochemistry indicated mature B lymphocytes, with some cells differentiating into plasma cells, leading to a final diagnosis of thymic MALT lymphoma.

In the present case, although a simultaneous diagnosis of pulmonary MALT lymphoma was made, the process of obtaining the final diagnosis was highly complex. The histopathological results of the lesion in the lower lobe of the right lung did not reveal the typical features of MALT lymphoma, and the final diagnosis of pulmonary MALT lymphoma was made in conjunction with a molecular examination. The diagnosis of lymphoma can be supported by clonality assessments because all cells of malignancies have a common clonal origin. A PCR gene scan is necessary to confirm monoclonality, which helps in obtaining the final diagnosis in patients with lymphoma [9, 10]. In our case, clonal IGH genes of PCR gene scan were detected in the thymic and lung foci. However, we did not find MALT lymphoma-related chromosomal abnormalities, including trisomy 3, MALT1 break apart, and IGH break apart, in the thymic and lung lesions by FISH. Thus, our MALT lymphoma had ongoing somatic mutations based on the analysis of IGH genes in the tumor cells, but there were no cytogenetic abnormalities such as IGH chromosome break or fusion with other chromosomes. Several chromosomal abnormalities are related to the occurrence and development of MALT lymphoma, involving chromosomes 3, 14, 11, and 18. Within these chromosomes, t(11;18) (q21; q21) is considered to be specific for MALT lymphoma and results in apoptosis inhibitor 2 (API2)–MALT1 fusion [11]. This mutation has been observed in 30% of MALT lymphomas of the lung but is rare or absent in those in the thyroid and salivary glands [12]. Satoru Kominato et al. detected chromosomal aberrations of 14 thymic MALT lymphomas; 50% of cases showed trisomy 3, while 7.1% showed trisomy 18, and none of the cases showed MALT1- or IGH-associated gene abnormalities, including t(11;18). The pattern of chromosomal aberrations in thymic MALT lymphomas was similar to those of autoimmune-associated MALT lymphomas of the thyroid and salivary glands [13]. Salivary gland and thyroid MALT lymphomas are associated with SS and Hashimoto’s disease, respectively; thus, the API2-MALT1 fusion may not play a genetic role in autoimmune-associated MALT lymphomas [14]. In this case, the patient was definitively diagnosed with MALT lymphoma with SS. Thymic MALT lymphoma usually progresses slowly, and to our knowledge, there have not been any reports describing cases of multiple organ metastases. However, the sequence of appearance of thymic and pulmonary MALT lymphomas is not entirely clear in our case. Nonetheless, considering their same molecular biology and cytogenetic characteristics, the thymus was more likely to be the first organ affected, and pulmonary MALT lymphoma or the presence of subhistological lymphoma may be the reason underlying cell recirculation that led to the accumulation of MALT within the lung [15–17].

In literature reviews, we found that the incidence of MALT lymphoma presenting simultaneously in two different organs is very low, and only three cases of combined thymic and pulmonary MALT lymphoma have been reported, including two patients with a history of SS [15–17]. MALT lymphoma is the most common lymphomatous type in SS patients [18]. Ying Liu et al. summarized 142 patients with SS complicated with MALT lymphoma from April 2010 to April 2020, indicating that the most common sites of MALT lymphoma are the parotid gland (77.5%), lung (14.8%), and thymus (5.6%) [19]. The main feature of SS is polyclonal lymphocytic infiltration, but the pathogenic mechanisms of the progression to lymphoma are not yet clear. M. Du et al. found that MALT lymphoma derives from postgerminal center memory B cells, possibly autoreactive B-cell clones, and that direct antigen stimulation may play an important role in the clonal expansion of low-grade MALT lymphoma [20].

Furthermore, histopathological examinations also revealed large amyloid deposits in lung MALT lymphoma. In B-cell lymphoma, amyloid deposits are found in only 2–4% of cases [21–23], and MALT lymphoma with amyloid deposition is even less common and can be found in organs such as the orbit, soft tissues, lungs, and breast [24–27]. The majority of reported cases of MALT lymphoma with amyloid deposition have exhibited an indolent clinical course [24]. In addition, such nodules of amyloid deposition may also relate to preexisting chronic inflammatory conditions or may be associated with autoimmune conditions such as SS, which produce excess immunoglobulin light chains that misfold and form deposits [28, 29].

Simultaneous thymic and pulmonary MALT lymphoma is relatively rare, and concomitant adenocarcinoma of the lung is even rarer. The incidence rates of primary pulmonary lymphoma and secondary pulmonary lymphoma are 0.5–1.0% and 0.5%, respectively, among lung malignancies, and MALT lymphoma is the most common type of pulmonary lymphoma [30]. However, non-small cell lung cancer accounts for 80~85% of lung cancer, and adenocarcinoma is the most common histological subtype of non-small cell lung cancer (50%) [31]. The published cases of simultaneous detection of pulmonary MALT lymphoma and lung adenocarcinoma are summarized in Table 1. There were nine cases, including the present case, of which five patients were males and four were females. Most patients were middle aged or elderly, and the median age was 68.5 years. Five patients did not present with specific symptoms, three of the male patients had a history of smoking, all nine patients underwent pulmonary lobectomy, and one patient underwent postoperative chemotherapy. All of these were typical cases of collision tumors, which differed from the progression of the same cell lineage to different malignant tumors; instead, these tumors originated from different lineages. The cause in the presented case was unknown, but the combination of these two tumors may have been due to the presence of common risk factors [32], or the appearance of one tumor may have altered the microenvironment and thereby promoted the development of the other tumor [33]. Genetic mutations are also a potential cause, but as far as the present study shows, there was no overlap in the genetic mutations common to thymic MALT lymphoma and lung adenocarcinoma [34, 35]. Translocation or point mutation of the ALK gene has been associated with driver oncogenesis in several tumors, such as non-small cell lung carcinoma, anaplastic large cell lymphoma, and inflammatory myofibroblastic tumors [36]. The MALT lymphoma and lung adenocarcinoma in our case were MALT1 and ALK break apart FISH negative. Nevertheless, some common signaling pathways, such as the NF-κB pathway, which exert anti-apoptotic action may be involved in their development [34, 37].

**Table 1:** The main characteristics of patients with lung MALT lymphoma and lung adenocarcinoma

Article	Age/sex	Symptoms	Smoking	Site	Surgery	Other treatment	Follow-up
Chanel et al. [38]	74/M	Febrile illness	> 10 years	LUL	Lobectomy with lymph node sampling	NA	NA
Akita et al. [39]	60/F	No symptoms	NA	LLL	Lobectomy	NA	NA
Ichihara et al. [40]	74/M	Cough and bloody sputum	NA	LAS	Lobectomy with	NA	NA
Kargi et al. [41]		Epigastric pain, nausea			Lymph node sampling		
	49/M	Loss of appetite	Heavy	LUL	Lobectomy	NA	NA
		Weight loss					
Jung et al. [33]	60/F	Health checkup	Never	RUL LMBL	Lobectomy with lymph node sampling	NA	NA
Zheng et al. [42]	81/M	No symptoms	NA	RML	Lobectomy	NA	9 months, died
Josefina et al. [43]	68/M	No symptoms	Heavy	RLL	Lobectomy with lymph node sampling	NA	NA
				RUL			
Sun et al. [3]	69/F	Headache, dizziness, and cough	No	RUL	Lobectomy with lymph node sampling	6 cycles of chemotherapy	12 months, alive

*M* male, *F* female, *LLL* left lower lobe, *LAS* left apicoposterior segment, *LUL* left upper lobe, *LMBL* left main bronchial lesion, *RUL* right upper lobe, *RML* right middle lobe, *RLL* right lower lobe, *NA* not available

Collision tumors are one of the most challenging tumor types to diagnose and treat, and their diagnosis is dependent on the identification of histopathological features in conjunction with the use of molecular examinations. There are no standards or guidelines for tumor grading, staging, treatment, and prognostic information. However, for collision tumors that originate from different cell lineages, independent staging and treatment can be used for each tumor separately. If the patient’s general condition permits, surgical resection is the treatment of choice, with radiotherapy only being indicated in patients with a single, small lesion, while systemic chemotherapy may be considered for large or disseminated cases [43]. Most studies have confirmed that patients with MALT have a relatively good prognosis regardless of the treatment modality. In the present case, the patient underwent total thymectomy and wedge resection of the lung without postoperative radiotherapy and exhibited satisfactory health at the 4-month follow-up.

The present case shows that MALT lymphoma must be considered when cystic space-occupying lesions of the thymus are found. Patients with pulmonary nodules also require further definitive diagnosis with supporting histopathological examinations. More molecular biology and cytogenetic examinations can also play an important role in the diagnosis of MALT lymphoma with atypical pathology. SS may be an important condition for the occurrence of MALT lymphoma in the thymus and lung. In addition, further evidence and case reports must be accumulated to elucidate the biological pathways and exact pathogenetic relationship of rare lymphomas and collision tumors to choose an optimal treatment.

## Data Availability

The relevant data and materials pertaining to this study are available upon request.

## Abbreviations

MALT: Mucosa-associated lymphoid tissue
SS: Sjögren’s syndrome
CT: Computed tomography
FISH: Fluorescence in situ hybridization
ALK: Anaplastic lymphoma kinase
MALT1: MALT lymphoma-associated translocation 1
Ig: Immune globulin
IGH: Immunoglobulin heavy locus
API2: Apoptosis inhibitor 2 gene
HE: Hematoxylin-eosin
M: Male
F: Female
LLL: Left lower lobe
LAS: Left apicoposterior segment
LUL: Left upper lobe
LMBL: Left main bronchial lesion
RUL: Right upper lobe
RML: Right middle lobe
RLL: Right lower lobe
NA: Not available
